# Dragon fruit (*Hylocereus undatus*) peel pellet as a rumen enhancer in Holstein crossbred bulls

**DOI:** 10.5713/ajas.20.0151

**Published:** 2020-08-21

**Authors:** Maharach Matra, Pajaree Totakul, Bounnaxay Viennasay, Burarat Phesatcha, Metha Wanapat

**Affiliations:** 1 Tropical Feed Resources Research and Development Center (TROFREC), Department of Animal Science, Faculty of Agriculture, Khon Kaen University, Khon Kaen 40002, Thailand; 2 Department of Agricultural Technology and Environment, Faculty of Sciences and Liberal Arts, Rajamangala University of Technology Isan, Nakhon Ratchasima 30000, Thailand

**Keywords:** Dragon Fruit Peel, Rumen Fermentation, Rumen Enhancer, Methane

## Abstract

**Objective:**

An experiment was conducted to assess the effect of dragon fruit peel pellet (DFPP) as a rumen enhancer of dry matter consumption, nutrient digestibilities, ruminal ecology, microbial protein synthesis and rumimal methane production in Holstein crossbred bulls.

**Methods:**

Four animals, with an average live-weight of 200±20 kg were randomly assigned in a 4×4 Latin square design to investigate the influence of DFPP supplementation. There were four different dietary treatments: without DFPP, and with 200, 300, and 400 g/h/d, respectively.

**Results:**

Results revealed that dry matter consumption of total intake, rice straw and concentrate were not significantly different among treatments (p>0.05). It was also found that ruminal pH was not different among treatments (p>0.05), whilst protozoal group was reduced when DFPP increased (p<0.01). Blood urea nitrogen and NH_3_-N concentrations were increased at 400 g of DFPP supplementation (p<0.01). Additionally, volatile fatty acid production of propionate was significantly enhanced by the DFPP supplementation (p<0.05), while production of methane was consequently decreased (p<0.05). Furthermore, microbial protein synthesis and urinary purine derivatives were remarkably increased especially at 400 g of DFPP supplementation (p<0.05).

**Conclusion:**

Plant secondary compounds or phytonutrients (PTN) containing saponins (SP) and condensed tannins (CT) have been reported to influence rumen fermentation. DFPP contains both CT and SP as a PTN. The addition of 400 g of DFPP resulted in improved rumen fermentation end-products especially propionate (C3) and microbial protein synthesis. Therefore, DFPP is a promising rumen enhancer and indicated a significant potential of DFPP as feedstuff for ruminant feed to mitigate rumen methane production.

## INTRODUCTION

Livestock production contributes to global warming by discharging greenhouse gas (GHG) either from manure management and enteric fermentation or feed production. The major GHGs are nitrous oxide (N_2_O), carbon dioxide (CO_2_) and especially methane (CH_4_) throughout the production process. Therefore, CH_4_ is the most important GHGs from the livestock [1].

The importance of ruminal ecology has been stated to the subsequent fermentation and livestock production efficiency [2]. Manipulation of ruminal fermentation has been reported using ionophores, antibiotics, chemical compounds, vaccination, and phytonutrients or plant secondary compounds [3]. Moreover, there is a need to utilize organic plant products that are used as livestock feed additives to improve the efficiency of feed use in ruminant production, and to decrease methane emission [4]. Natural products in tropical regions contain high to medium amounts of phytonutrients especially condensed tannins (CT) and saponins (SP), which have activity against protozoal populations and methane emission [5]. Previous studies have stated that the use of agricultural by-products and tropical plants especially fruit peel wastes containing phytonutrients have resulted in improved ruminal fermentation while decreasing production of methane [6,7]. Currently, plant secondary substances are an important area of research to replace feed additives in livestock [8]. The CT and SP are a group of phytonutrients of that demonstrate an ability to alter the rumen ecosystem, fermentation, reduces production of methane, and increases livestock production [9].

Among many tropical fruits, dragon fruit is abundant and its fruit peel is a waste from the fruit industry or fruit processing and is traditionally used as fertilizer. The peel has high amounts of phytonutrient [10]. Hence, dragon fruit can be considered as a good source of bioactive compounds for fermentation modification and health benefits. In addition, CT and SP are phytonutrients contained in dragon fruit peel and in many tropical plants [11]. In addition, Matra et al [12] studied *in vitro* fermentation by adding at 0%, 1%, 2%, 3%, and 4% dragon fruit peel powder containing 6.9% CT and 8.9% SP, resulting in improving ruminal fermentation and decreasing protozoal population and methane production with the 4% dragon fruit peel powder addition. However, there are limited studies on the impact of dragon fruit peel pellet (DFPP) containing CT and SP as a supplement on nutrient digestibility, rumen fermentation, and methane emission. Therefore, the study objective was to explore the effect of DFPP as a rumen enhancer on dry matter (DM) consumption, nutrient digestibilities, ruminal ecology, microbial protein synthesis and methane emission in Holstein crossbred bulls.

## MATERIALS AND METHODS

### Animal care

All Holstein crossbred bulls involved are managed and cared for according to the protocols of the Animal Care and Use Committee of Khon Kaen University and carried out by the Institute of Animals for Scientific Purpose Development (IAD), Thailand (record no. U1-06878-2560).

### Animal management

The experimental animals were injected with vitamin AD3E and drenched with Ivermectin (1 mL/50 kg live-weight [LW]; Merck Co., Inc., Kenilworth, NJ, USA) before imposing the respective treatments. They were housed in individual pens with clean drinking water and mineral blocks were made available freely.

### Preparing of dragon fruit peel pellet

Fresh dragon fruit peels were obtained from fruit canning factory. They were chopped and sun-dried approximately 2 or 3 days. Dried dragon fruit peels were processed by grinding to the length of 1 mm sieve (Cyclotech Mill, Tecator, Höganäs, Sweden). The powder was mixed with other components (Table 1) to form a pellet by using Ryuzo-kun pelleter (Kakiuchi Co., Ltd., Nankoku, Kochi, Japan).

### Experimental design and dietary treatments

Four Holstein crossbred bulls with an average LW of 200±20 kg were used. The experimental design was a 4×4 Latin square design with four different treatments (0, 200, 300, and 400 g/h/d DFPP, respectively). Animals were subjected to their dietary treatments during four periods consisting of 21 days per period; the first 14 days were used for diet adaptation and the last 7 days were used for data collection. The last 7 days, animals were moved to metabolism crates for total fecal and urine collection to study digestibility. Animals were offered rice straw as a roughage source *ad libitum* with 10% refusal and 0.5% concentrate of their body weight comprising 14.0% crude protein (CP), 27.6% neutral detergent fiber (NDF), and 18.2% acid detergent fiber (ADF) at 07.00 and 16.00 o’clock.

### Sample collection, preparation and chemical analyses

Urinary and fecal samples were collected during the last 7 days of each period when the animals were in metabolism crates. The feces were dried at 60°C for 2 or 3 days then were ground (Cyclotech Mill, Tecator, Höganäs, Sweden) and used for nutrient digestibilities. The concentrate, rice straw, dragon fruit peel and feces were chemically assessed for DM, ash, CP, and fiber contents. The DFPP samples were measured for CT and SP content following the procedure of Wanapat et al [13].

The urinary allantoin was analyzed by the procedure of Chen et al [14]. Microbial purine absorbed corresponding to the purine derivatives (PD) excretion was evaluated based on their relationship according to Chen and Gomes [15] as follows:

$$\text{Y}=0.85\text{X}+(0.385{\text{W}}^{0.75})$$

where Y is the urinary PD excretion (mmol/d); X is the microbial purine absorbed (mmol/d); and W is BW of animal (g/kg BW^0.75^).

The microbial protein supply in grams per day were cal culated following to the equation:

Microbial protein supply (g/d)=(X×70)/(0.116×0.83×1,000)=0.727×X

where X is the urinary PD absorption (mmol/d), according the suppositions calculated by Chen and Gomes [15]. The microbial purine digestibility is 0.83 and the N concentration of purine is 70 mg N/mmol. The ratio of purine-N to total N in mixed rumen microbes is 11.6:100.

Efficiency of microbial N synthesis (EMNS) was calculat ed according to the following formula:

EMNS=microbial N (g/d)/DOMR

where DOMR is rumen digestible organic matter (OM) apparently fermented (assuming that rumen digestion was 650 g/kg OM of digestion in total tract, DOMR = DOMI×0.65, DOMI = digestible OM intake according to the method of ARC [16].

On the last day of each period, at 0 h before feeding and 4 h after feeding, rumen fluid was taken via a tube connected to a vacuum pump. Firstly, the rumen fluid samples were immediately determined for temperature and pH (HANNA Instruments HI 8424 microcomputer, Singapore). Secondly, rumen fluid of 1-mL was mixed with 10% formalin of 9-mL for measuring total direct counts of bacteria, protozoa and fungi zoospore according to the procedure of Galyean [17]. Thirdly, 45-mL rumen fluid were mixed with 1 M sulphuric acid of 5-mL to prevent the fermentation process and kept in plastic bottle, and then centrifuged at 3,000×g for 10 minutes. After the centrifugation, the samples were used for NH_3_-N analysis (Kjeltech Auto 1030 analyzer, Tecator, Hoganiis, Sweden) following the method of AOAC [18] and volatile fatty acid (VFA) analysis using high performance liquid chromatography (Model Water 600; UV detector, Millipore Crop, Cortland, NY, USA) according to Samuel et al [19] procedure. The methane production was estimated from VFA profiles following the method of Moss et al [20].

Blood samples at 0 and 4 h after feeding were collected (10 mL from the jugular vein) on the last days and then added with ethylenediaminetetraacetic acid. They were centrifuged for 20 min to separate plasma and stored at −20°C for blood urea nitrogen (BUN) analysis [21].

### Statistical analysis

All data were analyzed according to the procedure of general linear model following to SAS [22]. The results are presented as mean values with the standard error of the means. Differences among treatment means with p<0.05 and p<0.01 were reported as being statistically different. All treatment means were analyzed according to the equation:

$${\text{Y}}_{\text{ijk}}=\mu +{\text{T}}_{\text{i}}+{\text{C}}_{\text{j}}+{\text{R}}_{\text{k}}+{\text{e}}_{\text{ijk}}$$

where μ, over all sample mean; T_i_, effect of treatment i; C_j_, effect of treatment i at column j; R_k_, effect of treatment i at row k; e_ijk_, error; and Y_ijk_, the criteria under study, in treatment i, column j, row k.

Treatment means were determined by Duncan’s new mul tiple range test according the standard method of Steel and Torrie [23]. Additionally, orthogonal polynomials for diet responses were detected by linear, quadratic and cubic effects.

## RESULTS

### Chemical composition of experimental diets

Nutritive values of feeds are presented in Table 1. Concentrate was formulated to contain 14.0% CP, 27.6% NDF, and 18.2% ADF. Cassava chip (60% as fed) was used as an energy source. While, rice straw (3.05 CP, 71.3% NDF, and 55.5% ADF) was fed as a roughage source. Additionally, DFPP has 9.7% CP, 17.8% NDF, 14.5% ADF, with 6.2% CT and 8.1% SP, respectively.

### Dry matter consumption and nutrient digestibilities

Table 2 illustrates the information on DM consumption and nutrients digestibilities as affected by the DFPP supplementation. The DM consumption of total intake, rice straw and concentrate were not affected by the DFPP supplementations (p>0.05). Moreover, nutrient digestibilities (DM, OM, CP, NDF, and ADF) were not affected (p>0.05) by the DFPP supplementation.

### Ruminal ecology and blood metabolites

The effect of DFPP supplementation on ruminal ecology and BUN concentration in Holstein crossbreed bulls is shown in Table 3. The ruminal temperature and pH were not altered by the DFPP supplementation (p>0.05). The protozoa numbers at 0 h before feeding and 4 h after feeding were linearly (p<0.01) decreased by 69.5% and 65.7% and bacterial populations were enhanced when DFPP supplementation were increased up to 400 g (p<0.01). The DFPP supplementation levels did not affect the population of fungi (p>0.05). The DFPP supplementation significantly increased (p<0.01) the concentrations of NH_3_-N. Also, BUN at 0 h before feeding and 4 h after feeding were significantly increased (p< 0.01) by increasing DFPP supplementations (23.4% and 23.8%).

### Ruminal volatile fatty acid and methane production

The propionate concentrations at 0 h before feeding and 4 h post feeding were markedly increased (p<0.05) by the DFPP supplementation at 400 g/d when compared to the control group (11.8% and 12.2%). The concentration of acetate, butyrate and acetate to propionate ratio were significantly decreased by the DFPP supplementation (p<0.05). Total VFA were not influenced by the DFPP supplementation (p>0.05). The DFPP supplementations did not affect the methane concentration compared to the control, and DFPP supplementation at 400 g/d showed the lowest CH_4_ production of 9.0% at 0 h before feeding and of 10.0% at 4 h post feeding (Table 4).

### Urinary purine derivatives and estimated microbial protein synthesis

Urinary PD, allantoin absorption and excretion were affected by the DFPP supplementation (p<0.05). Moreover, EMNS and microbial protein supply were improved by increasing level of DFPP supplementation especially at 400 g (p<0.05; Table 5).

## DISCUSSION

### Dry matter consumption and nutrient digestibilities

The DM consumption was not affected by the DFPP supplementations. This could be due to the DFPP having no negative effect on the palatability of the diet. Ampapon et al [24] showed that DM consumption was not significantly different among groups of swamp buffalos fed different levels of mangosteen peel powder. Addition of plants containing phytonutrients into animal diets normally decreases digestion rate. It has been shown that nutrients’ digestibility were reduced by high intake of CT and SP level [25]. In this experiment, nutrients’ digestibility among treatments were similar. This could be due to the appropriate dose of CT and SP in DFPP, hence DFPP supplementation could be an alternative feed without a negative effect on nutrients’ digestibility. Similarly, Ampapon et al [26] revealed that the nutrients’ digestibility was not changed in the rambutan peel additional groups.

### Ruminal ecology and blood metabolites

Russell and Rychlik [2] reiterated the important role of pH in maintaining normal activities in the rumen environment. Under this study, ruminal pH was 6.50 to 6.63, these values were not changed by adding DFPP at all levels. Accordingly, ruminal pH should be at 6.2 to 7.2 for microbial activity [27]. The protozoal population were decreased when adding DFPP, which could be attributed by the presence of phytonutrients especially CT and SP contained in the DFPP. These compounds could damage some enzymes and proteins on the plasma membrane of protozoa, causing the protozoa to die [28]. This finding agrees with Patra and Saxena [29], who reported that the population of protozoa was decreased by plant extracts (CT and SP) in the diets. Additionally, Matra et al [12] also explained that the inclusion of dragon fruit peel powder in the feed decreased protozoal population in *in vitro* study. Ruminal NH_3_-N concentrations were increased from 16.1 to 22.0 mg/dL by the DFPP supplementation. This may be due to the greater protein digestion from DFPP (9.7% CP), concentrate (14.0% CP) and non-protein nitrogen in the rumen [30].

Blood urea concentrations correlated with the ammonia nitrogen concentration availability in the rumen. Byers and Moxon [31], demonstrated that the optimal range of BUN was from 10.0 to 15.0 mg/dL. Under this study, BUN concentrations were increased when the cattle were fed with 400 g/h/d of DFPP, which ranged from 11.0 to 14.4 mg/dL. The reason of increasing BUN concentration could be due to the increase of ammonia nitrogen concentration. Accordingly, Foiklang et al [32] revealed that BUN concentration was higher in the mangosteen peel powder and grape pomace powder supplemented groups.

### Ruminal volatile fatty acids and methane production

Under this experiment, adding levels of DFPP increased propionate whilst acetate, butyrate and acetate to propionate ratio were remarkably decreased. This may be attributed to when acetate reduced, propionate could be increased while propionate remained unaltered [33]. However, acetate inhibitory effect might decrease the acetate to propionate ratio and directed to inhibit ruminal methanogen [34]. Anantasook et al [35] stated that decreasing ratio of acetate to propionate related to the increase of propionate production from a biological conversion of hydrogen as a substrate. Furthermore, the ruminal VFA production differences may occur due to reduced fermentation rates resulting from the amount of tannins and/or SPs [36]. This finding aligns with the report of Piñeiro-Vázquez et al [37], who revealed that the propionate proportion was improved by 3% and 4% tannins extracted from Quebracho plant. Foiklang et al [30], who also found that the high propionate proportion was caused by reducing methane production due to the CT containing in grape pomace powder (12.3% CT).

The current study has shown that increasing the DFPP linearly reduced methane production (0 and 4 h post feeding). A reduction of methane from DFPP feeding could be due to the effect of the CT and SP. Accordingly, Hristov et al [38] and Gemeda and Hassen [39] also explained that the use of phytonutrient plants containing CT and SP had decreased the production of methane, decreased protozoal population and modified VFA in the rumen. Four possible functions of tannin on CH_4_ reduction have been proposed: i) it acts to reduce methanogen bacteria; ii) it decreases the protozoal population; iii) it acts to reduce fiber degradation; and iv) it decreases the acetate to propionate ratio [28]. Yang et al [40] showed that addition of tannic acid in beef cattle significantly reduced CH_4_ production.

### Urinary purine derivatives and microbial protein synthesis

Urinary excretion is regarded as a marker of microbial protein synthesis in the rumen [41]. PD excretion and absorption were different among levels of DFPP supplementation, especially DFPP at 400 g had the highest value. Microbial protein supply under this trial, exhibited a quadratic increase. This could possibly be due to microbial protein supply and EMNS which may be reported that ruminal NH_3_-N produced was used for microbial activity and growth [42]. Firkins et al [43] explained that the synthesis of rumen microbial proteins would result in more proteins supplied to the small intestine of ruminants.

## CONCLUSION

Supplementation of DFPP at 400 g/h/d resulted in an improvement of ruminal ecology, increasing VFA production especially propionate, microbial protein synthesis and reduction rumen methane production. Hence, DFPP could be a promising rumen enhancer and mitigate rumen methane production.

## Figures and Tables

**Table 1:** Ingredients and chemical composition of the experimental feedstuffs

Category	Concentrate	Rice straw	DFP	DFPP
Ingredients (% as fed)				
Cassava chip	60.0			-
Palm kernel meal	13.0			-
Dried brewers’ grains	12.0			-
Rice bran meal	9.0			-
Urea	2.0			1.0
Molasses	2.0			1.0
Salt	1.0			1.0
Sulfur	0.5			1.0
Mineral premix1)	0.5			1.0
Dragon fruit peel powder	-			90.0
Cassava starch	-			5.0
Chemical composition				
Dry matter (%)	87.5	90.6	90.4	86.5
	----------------------% dry matter---------------			
Ash	5.8	12.3	1.5	1.4
Crude protein	14.0	3.0	5.4	9.7
Neutral-detergent fiber	27.6	71.3	33.0	17.8
Acid-detergent fiber	18.2	55.5	29.6	14.5
Condensed tannins	-	-	6.9	6.2
Saponins	-	-	8.9	8.1

DFP, dragon fruit peel; DFPP, dragon fruit peel pellet.

1) Mineral premix (contains per kg): vitamin A 10,000,000 IU; vitamin D 1,600,000 IU; vitamin E 70,000 IU; Fe 50 g; Mn 40 g; Zn 40 g; Cu 10 g; I 0.5 g; Co 0.1 g; Se 0.1 g.

**Table 2:** Effect of dragon fruit peel pellet supplementation on dry matter consumption and nutrient digestibilities in Holstein crossbred bulls

Items	DFPP supplementation (g/h/d)				SEM	Contrast		
	
	0	200	300	400		L	Q	C
Rice straw								
kg/d	3.9	4.0	4.0	4.0	0.53	0.46	0.57	0.58
% BW	1.7	1.8	1.9	1.8	0.62	0.09	0.15	0.17
Concentrate								
kg/d	1.0	1.2	1.1	1.2	0.68	0.09	0.17	0.19
DFPP								
kg/d	0.0^a^	0.2^b^	0.3^c^	0.4^d^	-	-	-	-
% BW	0.0	0.1	0.1	0.2	-	-	-	-
Total DM intake								
kg/d	4.9	5.4	5.4	5.6	0.71	0.28	0.30	0.39
% BW	2.3	2.4	2.5	2.5	0.54	0.56	0.65	0.66
Nutrients digestibility (%)								
Dry matter	65.6	65.1	63.8	62.4	1.02	0.34	0.36	0.40
Organic matter	62.6	60.4	58.2	57.6	1.14	0.44	0.48	0.72
Crude protein	61.4	62.5	62.3	61.9	0.04	0.11	0.26	0.29
Neutral detergent fiber	54.3	54.5	52.6	52.4	0.05	0.22	0.31	0.35
Acid detergent fiber	48.4	47.7	47.2	46.8	0.08	0.25	0.36	0.39

DFPP, dragon fruit peel pellet; SEM, standard error of the mean; L, linear; Q, quadratic; C, cubic; BW, body weight; DM, dry matter.

a–d Means in the same row with different superscripts differed (p<0.05).

**Table 3:** Effect of dragon fruit peel pellet supplementation on rumen characteristics and blood metabolites in Holstein crossbred bulls

Items	DFPP supplementation (g/h/d)				SEM	Contrast		
	
	0	200	300	400		L	Q	C
Ruminal temperature (°C)								
0 h post feeding	38.6	38.7	38.5	38.5	0.13	0.45	1.00	0.45
4	38.4	38.7	38.4	38.6	0.12	0.82	0.76	0.08
Mean	38.5	38.7	38.4	38.6	0.11	0.68	0.90	0.11
Ruminal pH								
0 h post feeding	6.5	6.5	6.6	6.6	0.11	0.68	0.76	0.71
4	6.5	6.6	6.7	6.6	0.12	0.94	0.83	0.75
Mean	6.5	6.5	6.6	6.6	0.07	0.52	0.55	0.42
Ruminal microbes (cell/mL)								
Total bacteria (×10^9^)								
0 h post feeding	31.3^a^	32.5^b^	34.2^c^	35.7^d^	0.15	<0.01	0.43	0.56
4	32.4^a^	33.9^b^	35.2^c^	37.8^d^	0.07	<0.01	0.15	0.27
Mean	31.9^a^	33.2^b^	34.7^c^	36.8^d^	0.11	<0.01	0.22	0.49
Protozoa (×10^6^)								
0 h post feeding	8.2^a^	5.6^b^	4.5^c^	2.5^d^	0.17	<0.01	0.26	0.05
4	7.0^a^	5.5^b^	3.3^c^	2.4^d^	0.15	<0.01	0.12	0.07
Mean	7.6^a^	5.6^b^	4.0^c^	2.5^d^	0.13	<0.01	0.07	0.60
Fungi zoospores (×10^4^)								
0 h post feeding	1.4	1.5	1.4	1.5	0.08	0.16	0.79	0.22
4	1.5	1.4	1.4	1.4	0.09	0.16	0.84	0.43
Mean	1.4	1.4	1.4	1.4	0.07	0.77	1.00	0.88
NH_3_-N concentration (mg/dL)								
0 h post feeding	14.4^a^	15.5^b^	17.3^c^	20.4^d^	0.13	0.01	<0.01	0.37
4	17.8^a^	18.6^b^	21.9^c^	23.6^d^	0.09	<0.01	<0.01	<0.01
Mean	16.1^a^	17.1^b^	19.6^c^	22.0^d^	0.10	<0.01	<0.01	<0.01
BUN concentration (mg/dL)								
0 h post feeding	10.5^a^	11.4^b^	12.6^c^	13.7^d^	0.10	<0.01	0.12	0.37
4	11.5^a^	12.6^b^	13.7^c^	15.1^d^	0.13	<0.01	0.40	0.64
Mean	11.0^a^	12.0^b^	13.2^c^	14.4^d^	0.11	<0.01	0.19	0.86

DFPP, dragon fruit peel pellet; SEM, standard error of the mean; L, linear; Q, quadratic; C, cubic; BUN, blood urea nitrogen.

a–d Means in the same row with different superscripts differed (p<0.05).

**Table 4:** Effect of dragon fruit peel pellet supplementation on ruminal volatile fatty acids production and methane production in Holstein crossbred bulls

Items	DFPP supplementation (g/h/d)				SEM	Contrast		
	
	0	200	300	400		L	Q	C
VFA productions (mol/100 mol)								
Acetate								
0 h post feeding	66.2^a^	65.4^b^	65.1^b^	64.4^c^	0.11	0.03	0.73	0.74
4	67.2^a^	66.1^b^	66.0^b^	65.0^c^	0.06	0.01	0.64	0.64
Mean	66.7^a^	65.7^b^	65.6^b^	64.7^c^	0.08	0.04	0.25	0.36
Propionate								
0 h post feeding	24.7^a^	25.8^b^	27.1^c^	28.0^d^	0.10	0.03	0.43	0.30
4	26.0^a^	27.0^b^	28.7^c^	29.6^d^	0.07	0.02	0.39	0.39
Mean	25.3^a^	26.4^b^	27.9^c^	28.8^d^	0.08	0.03	0.30	0.31
Butyrate								
0 h post feeding	9.1^a^	8.9^b^	7.9^c^	7.6^d^	0.09	0.03	0.86	0.80
4	6.9^a^	7.0^b^	5.4^c^	5.4^c^	0.07	0.02	0.08	0.09
Mean	8.0^a^	8.0^a^	6.6^b^	6.5^b^	0.06	0.04	0.71	0.78
Total VFA (mmol/L)								
0 h post feeding	107.2	106.5	106.3	106.9	0.09	0.24	0.35	0.38
4	110.3	110.4	112.1	111.6	1.05	0.36	0.42	0.46
Mean	108.8	108.5	109.2	109.3	1.04	0.27	0.39	0.45
Acetate/propionate ratio	2.7^a^	2.5^b^	2.4^c^	2.3^d^	0.04	0.01	0.62	0.67
Methane production (mM/L)								
0 h post feeding	26.7^a^	25.9^b^	25.0^c^	24.3^d^	0.10	0.01	0.47	0.47
4	25.9^a^	25.1^b^	23.9^c^	23.3^d^	0.07	0.02	0.51	0.50
Mean	26.3^a^	25.5^b^	24.5^c^	23.8^d^	0.06	0.01	0.23	0.20

DFPP, dragon fruit peel pellet; SEM, standard error of the mean; VFA, volatile fatty acids; L, linear; Q, quadratic; C, cubic.

a–d Means in the same row with different superscripts differed (p<0.05).

**Table 5:** Effect of dragon fruit peel pellet supplementation on urinary purine derivatives and microbial protein synthesis in Holstein crossbred bulls

Items	DFPP supplementation (g/h/d)				SEM	Contrast		
	
	0	200	300	400		L	Q	C
Urinary purine derivatives (mmol/d)								
Allantoin absorption	54.8^a^	79.5^b^	81.4^b^	85.6^b^	1.82	<0.01	0.05	0.41
Allantoin excretion	66.9^a^	88.6^b^	93.2^b^	98.4^b^	0.63	<0.01	0.04	0.26
Microbial protein supply (g N/d)	48.6^a^	64.4^b^	67.7^b^	71.5^b^	0.54	<0.01	0.04	0.26
EMNS (g N/kg of OMDR1))	18.4^a^	23.3^b^	23.6^b^	24.1^b^	0.25	<0.01	0.05	0.39

DFPP, dragon fruit peel pellet; SEM, standard error of the mean; EMNS, efficiency of microbial nitrogen supply.

1) OMDR (kg), 65% of organic matter digestible in total tract.

a,b Means in the same row with different superscripts differed (p<0.05).
